# Knowledge graph as an adaptive cognitive scaffolding: enhancing learning outcomes in CLIL-based international trade practice course under the OBE framework

**DOI:** 10.3389/fpsyg.2026.1802856

**Published:** 2026-05-22

**Authors:** Xiaoqin Yin, Fang Chen

**Affiliations:** 1 School of Foreign Languages, Hangzhou City University, Hangzhou, Zhejiang, China; 2 College of Education, Zhejiang University, Hangzhou, Zhejiang, China; 3 Hangzhou Collaborative Innovation Institute of Language Services, Hangzhou, Zhejiang, China

**Keywords:** adaptive cognitive scaffolding, CLIL, cognitive load, knowledge graph, OBE

## Abstract

Driven by the digital transformation of higher education, innovative instructional methodologies in Content and Language Integrated Learning (CLIL) are pivotal for developing interdisciplinary foreign language professionals. To address the dual cognitive load inherent in CLIL environments, this study proposes a Knowledge Graph (KG)-driven adaptive scaffolding framework and evaluates its effectiveness within an Outcome-Based Education (OBE) paradigm. Adopting an educational design research methodology, we implemented a 16-week intervention targeting 51 undergraduate English majors at a Chinese application-oriented university. To assess the model’s efficacy in promoting disciplinary and linguistic gains, the study utilized a multi-dimensional evaluation matrix and a normalized Course Objective (CO) attainment matrix aligned with the professional accreditation standards. Quantitative analyses revealed substantial improvements in domain-specific literacy (overall CO attainment = 0.78), with particularly notable performance in disciplinary knowledge (CO1 = 0.81) and language proficiency (CO2 = 0.82). However, a “knowledge-action gap” persisted in the development of practical skills (CO3 = 0.73). These findings suggest that while KG demonstrates exceptional strength in conceptual scaffolding, additional immersive simulations are required to cultivate high-order procedural knowledge. By addressing these cognitive and practical dimensions, the study provides a scalable framework for integrating KG-driven adaptive scaffolding into New Liberal Arts curricula, while also identifying the limits of its scaffolding approach in fostering practical expertise.

## 1 Introduction

The global higher education landscape is undergoing a significant digital paradigm shift, a movement catalyzed in China by the strategic incorporation of “educational digitalization” into national policy frameworks. As noted by the Ministry of Education, digital transformation has become the primary catalyst of education change worldwide (Huai, 2023). Under the “New Liberal Arts” initiative, CLIL has been established as the core pedagogical approach for cultivating interdisciplinary professionals who excel in both professional expertise and linguistic proficiency (Dalton-Puffer et al., 2024; Trinh et al., 2025; Bower et al., 2020). Nevertheless, the implementation of Content and Language Integrated Learning (CLIL) in interdisciplinary courses, like International Trade Practice, still faces challenges presented by the enduring “cognitive bottleneck.”

Empirical evidence suggests that the traditional CLIL environment often induces a dual cognitive strain (Jiang et al., 2023) where intrinsic load from complex disciplinary concepts like “Incoterms 2020” intersects with the extraneous load of processing these concepts in a second language (Sweller, 2023; Sweller, 2024). While OBE aims to mitigate these issues through goal-oriented design, its implementation in business-oriented CLIL frequently encounters challenges due to the fact that the course contents, learning resources, and assessment data remain semantically disconnected. This lack of a data-driven feedback loop prevents the realization of a true OBE closed-loop, as instructors struggle to map real-time linguistic and disciplinary mastery back to specific COs.

KG technology offers a transformative solution that avoids all these pitfalls by externalizing implicit disciplinary knowledge into structured, navigable, and visualized semantic networks. Unlike static repositories, KGs function as adaptive cognitive scaffolds that effectively address the “expertise reversal effect” through temporary support to novices while minimizing redundancy for advanced learners. By illustrating logical relationships and semantic connections among data, KGs enable students to allocate their cognitive resources toward advanced synthesis tasks rather than the rote memorization of fragmented information.

Within the Chinese academic landscape, scholars have begun exploring KG integration through multimodal and digital intelligence technologies. Recent studies have examined the synergy between KGs and Large Language Models (Chen et al., 2025; Luo and Zhang, 2023), empirical studies on KG-enabled pedagogy (Wang et al., 2025; Zhou et al., 2025; Yang et al., 2026), and the construction of specialized KGs for foreign language instruction (Ai et al., 2024; Zhao and Cheng, 2023). However, as noted by Wang et al. (2025), much of this domestic research remains confined to conceptual modeling or isolated resource construction. Crucially, there is a distinct paucity of empirical research investigating how KG-based frameworks can specifically enhance the OBE closed-loop within business-oriented CLIL contexts. Current domestic literature offers the “scaffolding” (Sun and Wu, 2026) but lacks the longitudinal, quantitative evidence required to prove that such digital architectures actually facilitate the transition from rote memorization to high-order synthesis in interdisciplinary settings.

To address these gaps, this study proposes an adaptive KG-driven adaptive scaffolding model and evaluates its effectiveness through a longitudinal case study under a synergistic theoretical framework integrating OBE, Cognitive Load Theory, and Adaptive Scaffolding. Beyond providing a procedural description, the paper critically examines how the framework’s structural components resolve existing theoretical gaps, proposing a scalable and technologically advanced model for developing interdisciplinary “New Liberal Arts” curricula.

## 2 Theoretical framework

### 2.1 CLIL within outcome-based education

CLIL is a pedagogical approach that integrates instruction in non-language subjects with learning in a second language (L2), emphasizing content mastery while utilizing language as a supportive tool for achieving this end (Dalton-Puffer et al., 2024). CLIL necessitates a delicate balance between subject-specific knowledge and linguistic competence. However, current research fields mainly follow the language-focused, content-supported approach (Dalton-Puffer et al., 2024; Lo, 2024; Li and Huang, 2022). This approach poses significant challenges when applied to interdisciplinary subjects, which prioritize specialized content while using language as a supportive tool.

OBE, characterized by reverse design and forward implementation, is one of the de-facto standards for modern educational system (Syeed et al., 2022). OBE ensures precise alignment between instructional design and learning objectives, forming a closed loop of Outcome-oriented—Design—Implementation. Recent studies feature in moving toward precision assessment (Yao et al., 2025; Xu et al., 2024), competency-driven AI integration (Pradeesh et al., 2025), and learner-centric flexibility (Yasmin and Yasmeen, 2021; Sullivan et al., 2025).

When integrating CLIL within an OBE framework, a critical challenge emerges: while OBE excels at establishing macro-level Course Objectives (COs) and precision assessments (Yao et al., 2025; Xu et al., 2024), it often fails to elucidate the micro-level cognitive processes learners must undertake to achieve those outcomes, failing to account for the cognitive friction where trade logic and L2 terminology compete for limited working memory resources. In domains like International Trade Practice, learners must simultaneously navigate intricate trade logic and professional L2 discourse. This dual-layered cognitive demand often results in a scenario where intended learning objectives are defined, but the procedural steps required to achieve them remain fragmented and obscure due to insufficient instructional coherence.

### 2.2 Cognitive load theory and zone of proximal development

The failure to achieve OBE closed-loops in CLIL environments can be explained primarily through the lens of Cognitive Load Theory and Zone of Proximal Development. Both theories emphasize learners’ cognitive limitations and converge on the central objective of optimizing the learning process and improving its effectiveness.

Cognitive load refers to the mental pressure or burden exerted on a learner’s cognitive system while performing a specific task (Karabay and Meşe, 2025). According to the cognitive load theory, knowledge is categorized into “primary knowledge” which can be acquired effortlessly and naturally (e.g., learning a native language) and “secondary knowledge” which requires explicit instruction and imposes a substantial cognitive load (e.g., mathematics and programming) (Chen et al., 2021). This distinction holds crucial implications for designing educational frameworks in CLIL settings, where students must grapple with secondary knowledge (e.g., trade regulations). Such scenarios underscore the importance of effectively managing cognitive load to preclude students from encountering learning inefficiencies caused by cognitive overload (Gkintoni et al., 2025). The cognitive load theory (CLT) suggests that presenting redundant instruction to higher prior knowledge learners imposes a higher cognitive load resulting in the expertise reversal effect (Sweller et al., 2011). This phenomenon occurs when instructional supports beneficial to novices become obstacles for high-proficiency students, as they face the challenge of processing redundant information which their mental structures already processed (Faber et al., 2024). As learners’ knowledge and skill levels develop, the guidance provided to facilitate the effective and efficient acquisition of knowledge or the elicitation of learning processes should be gradually reduced. This gradual reduction is referred to as the “Guidance Fading Effect” or “Effects of Fading Support” (Renkl, 2012).

Zone of Proximal Development (ZPD) refers to an optimal learning environment in which tasks are structured to be neither overly challenging nor unduly simplistic (Vygotsky, 1978). This balance prevents states of boredom or confusion, both of which can result in distraction, frustration, and diminished motivation (Miranda et al., 2025). Optimal conditions within the ZPD are highly individualized and dynamic, evolving in response to each learner’s unique characteristics and the specific educational context (Miranda et al., 2025). ZPD addresses the gap between a learner’s actual ability to solve problems independently and their potential level of development achievable through guidance or collaboration with more capable individuals (Liu et al., 2024). This framework highlights the importance of social interaction and scaffolding support in fostering cognitive growth, making it a dynamic concept that varies across learners and contexts.

In summary, ZPD offers a theoretical framework for establishing appropriate learning goals, whereas CLT defines constraints on the pathways to achieve those goals. Effective teaching must simultaneously satisfy two conditions: learning tasks should be positioned within the ZPD (challenging yet attainable), and cognitive load must remain within the processing limits of working memory, avoiding overload due to flawed institutional design.

Modern educational technologies have increasingly integrated the principle of ZPD and CLT to enhance learning efficiency. These technologies aim to accurately define students’ ability boundaries based on the ZPD while regulating the complexity of scaffolding content to prevent cognitive overload. Scaffolding is an instructional method in which educators offer guided support to learners, reducing task complexity and enabling them to achieve task completion effectively (van Nooijen et al., 2024). Recent studies underscore the need to transition from static scaffolding to adaptive scaffolding with fading mechanisms to optimize learning efficiency (Karabay and Meşe, 2025). Adaptive scaffolding modifies the level of support and real-time feedback based on learners’ developing proficiency. This approach ensures that cognitive load stays within a manageable range throughout the learning process while maintaining an appropriate level of challenges within the ZPD.

### 2.3 Knowledge graph-driven adaptive scaffolding

To address the CLIL-OBE gap and alleviate cognitive load, this study proposes KGs as a technological implementation of adaptive scaffolding. KGs are architected as directed labeled graphs, where domain-specific nodes are inextricably linked by relational edges, representing a sophisticated evolution from static concept maps to dynamic, AI-integrated infrastructures (Chaudhri et al., 2022; Zhu et al., 2024; Bhatti et al., 2023). In the domain of International Trade Practice, nodes represent complex entities (e.g., bill of exchange/draft, promissory note, cheque), while edges explicate the interstitial relationships between them (e.g., instruments of payment) (Hao et al., 2021). Previous research has validated the efficacy of KGs in various domains, including personalization (Ma et al., 2025; Ezaldeen et al., 2022; Troussas and Krouska, 2023), intelligent tutoring (Li et al., 2024; Jing et al., 2020), resource development (Liang et al., 2024; Ma, 2022), mobile learning (Zhao et al., 2022) and synergy with Large Language Models (Ma et al., 2025; Chen et al., 2025). However, a critical limitation exists that most KG applications are confined to mono-disciplinary fields like computer science (Nafa et al., 2022), cybersecurity (Agrawal et al., 2022), clinical microbiology (Liu et al., 2025; Faber et al., 2024), automatic control (Ren et al., 2025), English language learning (Wu and Jia, 2022; Lou, 2025; Sun and Wu, 2026), and ancient Chinese poetry (Qu et al., 2024). Their potential as semantic scaffolding tools for interdisciplinary contexts remains under-explored.

In contrast to traditional scaffolding, which typically regards language and content as parallel elements, KG-driven scaffolding resolves the cognition-language dualism by mapping them onto a unified semantic network via two key mechanisms. First, by transforming implicit expert logic into explicit nodes and edges, the KG not only facilitates learning but also effectively offloads the linguistic decoding burden to the visual–spatial system. This redistribution expands the learner’s effective ZPD without surpassing the CLT threshold. Second, KGs facilitate adaptive scaffolding fading by gradually replacing explicit nodes with implicit conceptual links as learners progress from novice to competent states (Tang et al., 2026). This dynamic adaptation provides personalized feedback and path recommendations (Ma et al., 2025), mitigating the expertise reversal effect and enhancing cognitive efficiency throughout the learning process.

The effectiveness of teaching methods hinges on appropriate scaffolding, which demands educators to strike a pedagogical balance between insufficient scaffolding and excessive support. Insufficient scaffolding fails to alleviate the cognitive load, while excessive scaffolding can increase extraneous load. Moreover, overly excessive scaffolds may result in learners “offloading” their cognitive efforts onto the system, leading to homogenized ideas and diminished deep conceptual understanding (Bozkurt et al., 2024). The objective of scaffolding has evolved beyond simply making learning easier; it now aims to leverage AI to dynamically adjust challenge levels, ensuring learners maintain high engagement and deep cognitive processing. By analyzing unobtrusive data points, such as students’ knowledge level, mental capacity, stress levels, and performance outcomes, the KG-driven adaptive scaffolding model initiates tailored interventions aligned with the students’ support needs (Hennings et al., 2021; Cohn et al., 2025).

In summary, the integration of these theories establishes a cohesive instructional framework. OBE defines the navigational target (expected outcomes), while ZPD and CLT delineate the navigational boundaries (balancing challenge and cognitive capacity). Additionally, the KG-driven scaffolding functions as the navigational engine providing adaptive instructional support. This integration ensures that the “reverse design” methodology of OBE is complemented by a “forward implementation” strategy aligned with human cognitive architecture, effectively completing the loop of outcome-oriented interdisciplinary teaching.

## 3 Research design

### 3.1 Research objectives

International Trade Practice stands as a fundamental elective course designed for English majors specializing in business. The course focuses on teaching students the comprehensive process of import–export contracts, using English as the medium of instruction. Despite the relatively straightforward nature of individual knowledge points, students often face the dual challenge of mastering complex trade operations, while simultaneously engaging in linguistic processing. Traditional pedagogical approaches frequently struggle with fragmented content, scattered resources, and insufficient personalized scaffolding. The rapid expansion of artificial intelligence, coupled with evolving educational standards, calls for a transition to digitally intelligent talent cultivation through the adoption of innovative teaching strategies (Cai, 2023). In response to these challenges, this research proposes a KG-based CLIL framework. By utilizing KGs as adaptive cognitive scaffolding, the study aims to achieve three key objectives:

1. Develop a comprehension KG that integrates disciplinary expertise with international trade patterns, making implicit expert logic explicit through a structured visual format.
2. Create a customized learning system that employs the KG to provide adaptive and differentiated learning paths, thereby enhancing the effectiveness of the CLIL approach.
3. Establish a multidimensional evaluation system to objectively assess teaching outcomes, ensuring a robust feedback loop for continuous improvement in talent cultivation.

### 3.2 Participants

The study was conducted at a regional application-oriented university in Eastern China undergoing digital transformation. The participants consisted of 51 Business English majors enrolled in the International Trade Practice elective course during the 2024–2025 academic year. These students demonstrated upper-intermediate English proficiency as evidenced by achieving the Test for English Majors-Band 4 (TEM-4)^1^ certification or an equivalent qualification. Prior to enrolling in the course, students had completed foundational bilingual or English-medium courses in economics and management, including Principles of Economics (Bilingual), Fundamentals of Management (English), and International Marketing (Yin and Chen, 2026). However, their prior knowledge of specific international trade operations was minimal. The study took place in the university’s Cross-border Business Innovation Lab, equipped with laptops and POCIB^2^ simulation software.

### 3.3 Research methodology

**Figure 1:**
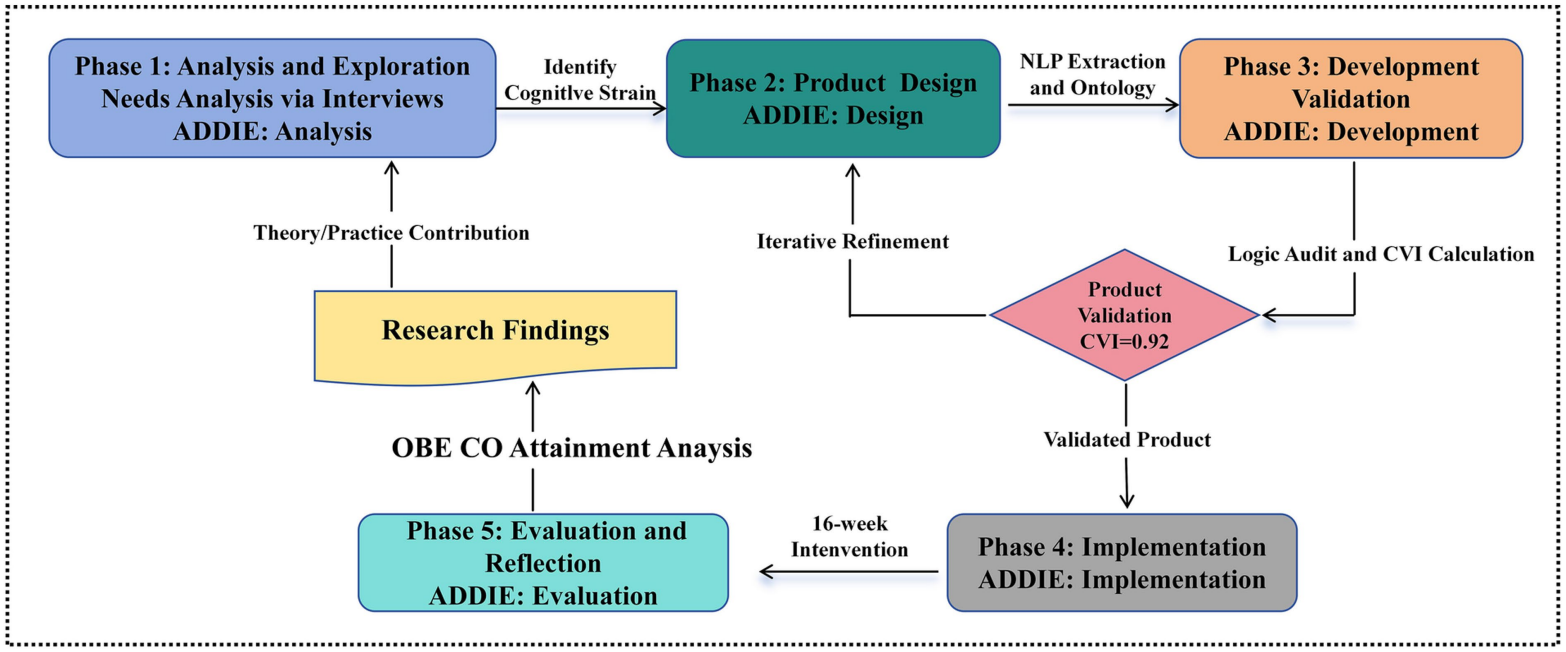
Flowchart of EDR framework.

Given the ecological constraints of authentic classroom settings, this study employs a quasi-experimental, single-group design within an educational design research (EDR) framework (Jacobsen and McKenney, 2024). While the research adheres to the overarching EDR logic of “analysis/exploration, design/construction, and evaluation/reflection”, the development of the pedagogical product specifically integrates the ADDIE (need analysis, design, development, implementation, and evaluation) model to ensure a systematic instructional design (Figure 1). To mitigate the inherent limitations of lacking a control group, the study utilizes longitudinal attainment tracking and course objectives (COs) attainment data derived from the OBE accreditation system. This methodology ensures that the observed improvements align directly with predefined proficiency benchmarks rather than merely reflecting general temporal progression. The research unfolded through five iterative phases of EDR:

*Phase 1*: Needs Analysis and Exploration: Through qualitative interviews and classroom observations, it was identified that students struggled with the dual cognitive strain of mastering complex trade logic while simultaneously processing disciplinary English terminology. This phase established the functional requirements for a visual and adaptive scaffolding tool aiming at externalizing expert logic and disciplinary English terminology and reducing cognitive strain.

*Phase 2*: Product Design: The target product, a KG-driven adaptive scaffolding model, was conceptualized. The technical development consisted of three steps: knowledge extraction, ontology construction, and visualization integrated with storage.

*Phase 3*: Development and Validation: The framework underwent a rigorous expert consultation and iterative logic audit to ensure its validity as a pedagogical tool.

*Phase 4*: Implementation: The KG-based intervention was implemented over a 16-week semester using a “blended-scaffolding” approach. This approach facilitated both synchronous classroom instruction and asynchronous autonomous learning, improving flexibility and engagement.

*Phase 5*: Evaluation and Reflection: Following the EDR evaluation cycle, the effectiveness of the KG-driven adaptive scaffolding model was assessed using OBE CO attainment calculations. These results were derived from a weighted matrix combining formative assessments with summative evaluations, offering a multidimensional perspective of the product’s instructional impact. Specifically, all data collected in each stage of the iterative research process (e.g., recorded classroom intervention, semi-structured interviews with students, assessment scores) were sorted out. Then, corresponding adjustments were made to the model and teaching implementation plan based on the research conclusions, to gradually optimize the applicability and effectiveness of the KG-driven adaptive scaffolding model in CLIL teaching.

## 4 Implementation of the KG-based pedagogical reform

Traditional CLIL faced challenges such as fragmented resources and elevated extraneous cognitive load. The course KG purports to externalize expert logic and represents a structured and visualized outcome of course content. It achieves this through organizing and extracting key knowledge points, establishing a relational network among these points, and connecting them with course resources (e.g., unit courseware, videos, textbooks, and question bank). The KG demonstrates four prominent features: semantic networking of course content, accurate recommendations of learning resources, customized learning path recommendations, and real-time feedback with closed-loop optimization. By harnessing these features, the KG-based pedagogical reform reduces the cognitive load and promotes adaptive learning. To ensure a proper alignment between technological intervention and instructional needs, this study adhered to the EDR approach, operationalized through the five-phase ADDIE model. This systematic methodology ensures that the designed KG is not merely a technical innovation, but a rigorously validated pedagogical tool.

### 4.1 Product development: from needs analysis to expert validation

In the era of artificial intelligence, KG stands as a foundational technology pillar for promoting educational digitalization. By employing semantic networks characterized by structural rigor and relational depth, KGs tools are increasingly seen as essential for enhancing teaching and learning, ultimately driving the digital transformation of education (Yuan, 2023; Zhu et al., 2021). The development of the KG-driven adaptive scaffolding model progressed through the first three phases of the ADDIE framework:

*Step 1*: Analysis and Design: Initial qualitative semi-structured interviews revealed that students encountered a dual cognitive challenge: processing complex trade logic while simultaneously navigating domain-specific English terminology. Specifically, 78% of the interviewed students reported significant difficulties in synchronizing their understanding of intricate trade concepts, such as Letters of Credit and Incoterms, with decoding specialized English vocabulary. Interview transcripts further demonstrated a recurring “bottleneck effect,” wherein the linguistic processing demands drained students’ cognitive resources, hindering their ability to perform high-order analytical tasks aligned with the OBE framework requirements. One instructor observed, one instructor noted that “students often lose the thread of trade negotiations because they are stuck on the legal nuances of the vocabulary”. In response to these challenges, the KG was designed to externalize implicit expert reasoning and provide a structured representation of knowledge, thereby alleviating the cognitive load by decoupling conceptual logic from linguistic hurdles.

*Step 2*: Development and Technical Pipeline: The development phase involved a multi-stage semantic mapping process, encompassing knowledge extraction, ontology construction, and system integration.

- *Knowledge Extraction*: Entities were extracted from authorized textbooks and digital repositories using natural language processing (NLP) technologies (e.g., BERT, spaCy). This automated process was followed by manual refinement led by subject-matter experts to ensure accuracy.
- *Ontology Construction*: A total of 349 distinct entities and 123 directed edges were defined to establish logical dependencies and hierarchical relationships. Core relationships in the course content, such as preceding/sequent knowledge points, inclusion/exclusion, exam point associations, resource linkages, were extracted using a relationship classification model, rule-based templates, and NLP technology. Additionally, attributes of the knowledge points, including cognitive levels, difficulty coefficients, assessment types, were annotated through keyword matching and semantic similarity calculations.
- *Visualization and Storage*: Neo4j was employed to construct the core KG, enabling efficient Cypher-based query execution and interactive visualization. NetworkX was integrated for advanced graph structure analysis and metric computation. On the frontend, D3.js facilitated interactive graph exploration, while ECharts rendered clear mind maps and conceptual frameworks. Together, these technologies implemented a comprehensive full-stack graph computing solution that encompassed storage, algorithmic analysis, and visualization. The resulting graph was deployed on the Xueyin Online platform^3^. The knowledge graph integrates intelligent algorithms to synchronize terminal data across MOOC videos, course materials, and test banks, effectively externalizing implicit expert logic in the domain of global trade. The KG architecture is composed of two main components:
- *Nodes*: Representing 349 distinct entities, including knowledge points such as FOB, CFR, bills of exchange, promissory note, and insurance policy.
- *Edges*: Defining 123 directed labeled edges that capture the functional and logical dependencies between nodes. Examples include “Bills of exchange are a payment instrument” or “precedes” relationships in international trade.

**Figure 2:**
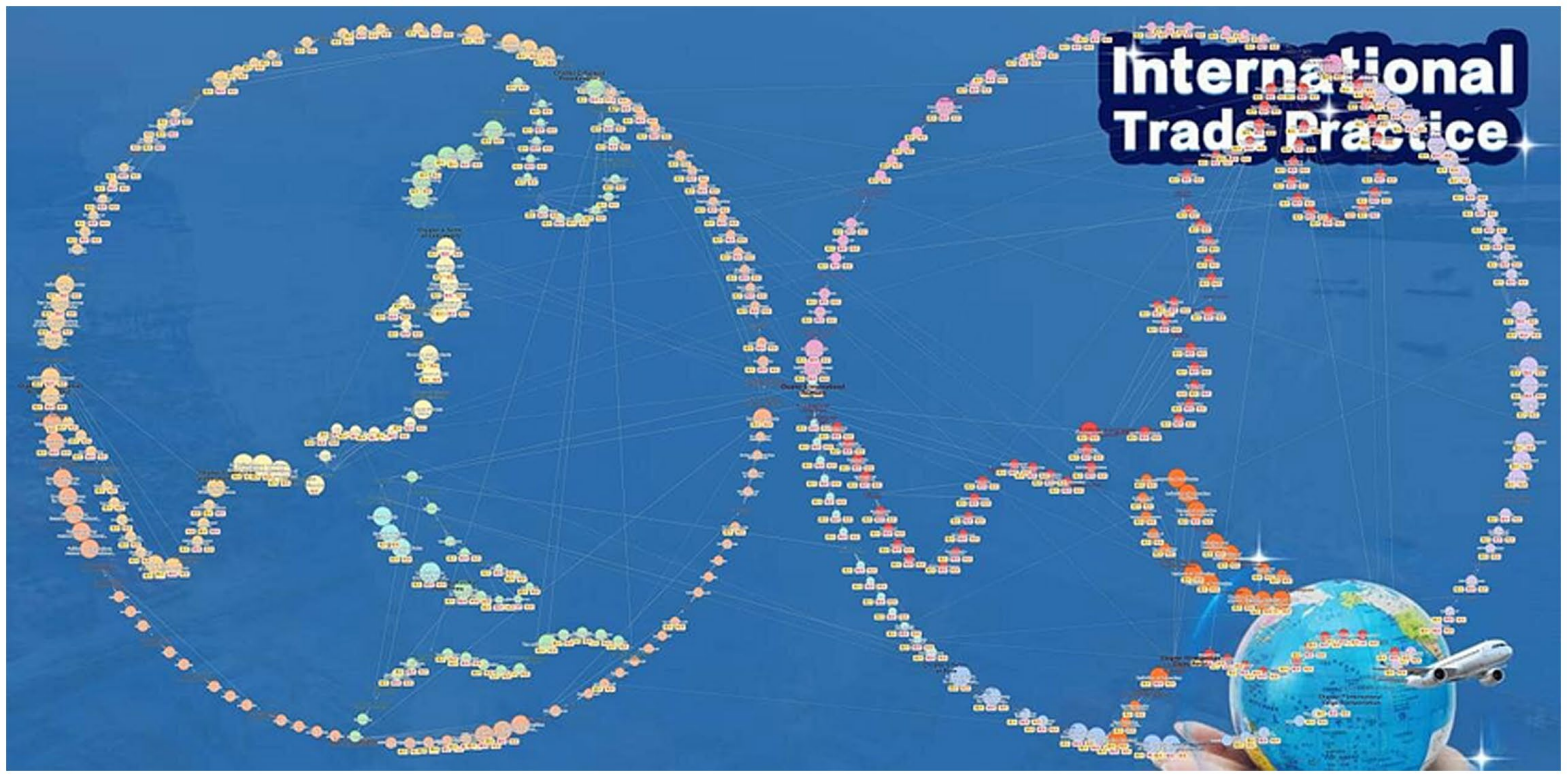
Knowledge graph of international trade practice.

International Trade Practice KG (Figure 2) transforms course content into an interconnected network, revealing the inherent operational structure of international trade activities. It also enables real-time synchronization of data across multiple terminals, including videos, courseware, and test banks. The KG-based framework encompasses a self-compiled textbook, 10 units of teaching courseware, together with 43 videos which run for 463 min, and a question bank that contains 315 items. This design allows users to seamlessly navigate between MOOC content and the question bank, such as linking challenging questions to relevant course sections or assessing supplementary practice questions aligned with specific MOOC modules. By streamlining access to contextual learning resources, the framework significantly enhances learning efficiency and depth.

*Step 3*: Development and Validation: To ensure the pedagogical and structural robustness of the model, the framework underwent a rigorous validation process incorporating both content validity index^4^ (CVI) (Polit and Beck, 2006) and iterative logic audits. A panel of five experts comprising three disciplinary experts and two instructional designers independently evaluated the KG based on five key criteria of node validity, relational logic, structural integrity, instructional usability, and resource mapping (Table 1). Experts rated each item on a 4-point Likert scale (1 = not relevant, 4 = highly relevant), with ratings of 3 and 4 considered endorsements (Tables 2, 3). The CVI was calculated using the following formulas.^5^

$$I-\mathrm{CVI}=\frac{A}{N}$$

$$S-\mathrm{CVI}/\mathrm{Ave}=\text{Average}\;(\frac{\begin{array}{c} I-\mathrm{CVI}1+I-\mathrm{CVI}2\\ +I-\mathrm{CVI}3+\ldots I-\text{CVIN} \end{array}}{N})$$

**Table 1:** Expert evaluation form for KG-driven scaffolding model content validity.

Evaluation item	Assessment criteria	1 Not relevant	2 Somewhat relevant	3 Relevant	4 Highly relevant
Node Validity	Accuracy of semantic definitions for entities (e.g., FOB, L/C, Bill of Lading).	□	□	□	□
Relational Logic	Rigor of logical dependencies and hierarchical edges (e.g., “Precedes,” “Is subbranch of”).	□	□	□	□
Structural Integrity	Alignment of the KG hierarchy with the tiered Bloom’s taxonomy in OBE goals.	□	□	□	□
Instructional Usability	Alignment of the KG hierarchy with the tiered taxonomy in OBE goals.	□	□	□	□
Resource Mapping	Accuracy of the synchronization between KG nodes and MOOC/Textbook resources.	□	□	□	□

**Table 2:** Evaluation composition and course objectives.

Evaluation composition		Test item no.		CO1	CO2	CO3	Total score
Regular grade (40%)	In-class performance	\		25			25
	Xueyin Online test	\		10	30	35	75
	Total score			35	30	35	100
Final exam (60%)	Multiple-choice question	I	1	2			2
			2		2		2
			3	2			2
			4		2		2
			5			2	2
			6		2		2
			7			2	2
			8	2			2
			9	2			2
			10		2		2
			11	2			2
			12		2		2
			13			2	2
			14			2	2
			15	2			2
		Item total score		**12**	**10**	**8**	**30**
	Term translation	II	1		2		2
			2		2		2
			3		2		2
			4		2		2
			5		2		2
		Item total score		**0**	**10**	**0**	**10**
	Calculation	III	1	2		3	5
			2	1	1	3	5
			3	1	1	3	5
		Item total score		**4**	**2**	**9**	**15**
	Case analysis	IV	1	2	3	3	8
			2	2	3	2	7
		Item total score		**4**	**6**	**5**	**15**
	documents question	V	**3–4; 10–13**	12			12
			**1–2; 5–9; 14–15**			18	18
		Item total score		12	0	18	30
	Total score			**32**	**28**	**40**	**100**

The bold values represent the total score of each item supporting the relevant CO and the total score of each item. For example, 12 represents the total score of Multiple-choice question supporting the CO1; 10 represents the total score of Multiple-choice question supporting the CO2; 8 represents the total score of Multiple-choice question supporting the CO3; 30 represents the total score of Multiple-choice question.

**Table 3:** Summary of expert ratings and CVI calculation.

Dimensions	E1	E2	E3	E4	E5	Number of endorsed (3 or 4)	I-CVI
1. Node Validity	4	4	4	3	4	5	1
2. Relational Logic	4	3	3	4	4	5	1
3. Structural Integrity	3	4	3	4	3	5	1
4. Instructional Usability	4	4	3	3	2	4	0.8
5. Resource Mapping	3	3	4	4	2	4	0.8
S-CVI/Ave	0.92						

The quantitative data obtained from the expert panel were analyzed using the item-level content validity index (I-CVI) and the scale-level content validity index (S-CVI). As indicated in Table 4, the I-CVI for individual items ranged from 0.80 to 1.00. Notably, the experts reached unanimous agreement on the “Node Validity,” “Relational Logic,” and “Structural Integrity” of the KG (I-CVI = 1.00). In contrast, the scores for “Instructional Usability” and “Resource Mapping” were slightly lower (I-CVI = 0.80), primarily due to Expert 5’s recommendation to further subdivide the “document against payment” sub-nodes and adjust the MOOC videos accordingly. The overall S-CVI/Ave was computed at 0.92, which exceeds the professional threshold of 0.90 (Polit and Beck, 2006; Waltz et al., 2005), thereby confirming the model’s strong content validity and its suitability for instructional implementation.

**Table 4:** Quantitative analysis of the achievement of the CO.

CO	Evaluation section		Total supporting grade		Average score	supporting weightage	Weighted mean score	Grade rate	Overall supporting weightage	Degree of achievement	
			Formula	Score							
CO1	In-class performance	25%	(25*40%)	10.0	92.0	0.10	9.20	92.00%	0.30	0.28	0.81
	Xueyin online Test	10%	(10*40%)	4.0	90.0	0.04	3.60	90.00%	0.12	0.11	
	Multiple-choice question	10%	(12*60%)	7.2	10.16	0.60	6.10	84.67%	0.22	0.18	
	Calculation	4%	(4*60%)	2.4	1.42	0.60	0.85	35.50%	0.07	0.03	
	Case analysis	4%%	(4*60%)	2.4	3.32	0.60	1.99	83.00%	0.07	0.06	
	documents question	12%	(12*60%)	7.2	8.47	0.60	5.08	70.58%	0.22	0.15	
CO2	Xueyin online Test	30%	(30*40%)	12.0	90.0	0.12	10.80	90.00%	0.42	0.38	0.82
	Multiple-choice question	10%	(10*60%)	6.0	8.2	0.60	4.92	82.00%	0.21	0.17	
	Term Translation	10%	(10*60%)	6.0	6.18	0.60	3.71	61.80%	0.21	0.13	
	Calculation	2%	(2*60%)	1.2	1.98	0.60	1.19	99.00%	0.04	0.04	
	Case analysis	6%	(6*60%)	3.6	4.99	0.60	2.99	83.17%	0.13	0.10	
CO3	Xueyin online Test	35%	(35*40%)	14.0	90.0	0.14	12.60	90.00%	0.37	0.33	0.73
	Multiple-choice question	8%	(8*60%)	4.8	6.2	0.60	3.72	77.50%	0.13	0.10	
	Calculation	9%	(9*60%)	5.4	6.59	0.60	3.95	73.22%	0.14	0.10	
	Case analysis	5%	(5*60%)	3.0	4.24	0.60	2.54	84.80%	0.08	0.07	
	documents question	18%	(18*60%)	10.8	8.06	0.60	4.84	44.78%	0.28	0.13	
CO achievement rate	0.78										

In addition to quantitative scoring, qualitative feedback from the expert logic audit offered valuable insights for iterative refinement. Based on expert recommendations, 12 relational edges were restructured to more accurately represent the chronological dependencies in international payment sequences. Additionally. eight redundant nodes were pruned to maintain the “minimal spanning tree” architecture of the KG.

### 4.2 Implementation: from linear content to semantic navigation

In the contemporary CLIL context, digital technologies hold significant importance. However, the effectiveness of specific tools is intrinsically dependent on their compatibility with instructional environments and learner preferences (Hu et al., 2025). The application of KGs in smart education marks a paradigm shift from traditional flipped classroom and blended learning models. Conventional approaches mainly focus on the spatial and temporal reconfiguration of content, often deconstructing coherent knowledge into discrete and isolated elements, which is similar to disassembling a forest into unconnected leaves. Such fragmentation often fails to alleviate the cognitive friction caused by fragmented resources, resulting in learner disorientation and an increased cognitive burden. In contrast, the KG-based framework utilizes a “minimal spanning tree” architecture, providing pedagogical benefits such as visual navigation, AI-mediated inquiry, personalized learning path tracking, and real-time progress monitoring (Shi et al., 2020; Cai et al., 2025). This structural enhancement conceptual coherence and significantly minimizes the risk of student disorientation. Instead of focusing solely on instructional delivery (how to teach), the framework emphasizes the key dimensions of pedagogy: content architecture (what to teach), entry points (where to initiate), and proficiency benchmarks (what level to achieve) (Shi et al., 2020). As a result, the classroom environment transforms from a traditional teacher-student dyadic interaction into a dynamic triadic interaction system, incorporating teachers, students, and artificial intelligence.

The validated KG model was implemented during a 16-week semester within a Cross-border Business Innovation Lab. Following the principle of integrating theory and practice, the instructional sequence was organized into four distinct and structured stages:

*Step 1*: Pre-class AI-Guided Navigation: Students interacted with KG on the Xueyin platform to internalize the macroscopic structure of upcoming topics. Utilizing pre-test data, the platform’s algorithm analyzed the KG to generate a visualized, personalized learning trajectory, and pushed targeted MOOC videos to address specific areas of weakness in their understanding. The personalized suggestions and tailored content supported students in addressing their learning difficulties (Fidan and Gencel, 2022), while enabling them to understand and learn from their mistakes in real time. This approach ensured their progress remained within the ZPD (Crompton and Burke, 2023).

*Step 2*: In-class Case-Based Instruction: Informed by pre-class data analysis, instructors conducted case-based discussions and bilingual role-play activities (e.g., price negotiations), placing emphasis on addressing identified cognitive bottlenecks. During lectures, the KG is employed to dynamically demonstrate complex semantic connections, effectively linking theoretical concepts with practical applications in international trade scenarios. The teaching process follows outcome-orientation logic and integrates content and language teaching in all sessions, ensuring learners improve professional knowledge and bilingual communication ability simultaneously.

*Step 3*: Post-class Adaptive Scaffolding: The Xueyin Online learning platforms are accessible anytime and anywhere, providing students with on-demand support regardless of geographical location or time constraints. This ensures that all students, including those in remote areas, have access to quality education within their ZPD (Chang et al., 2023). Additionally, the KG-driven adaptive scaffolding model enhances learning personalization and engagement by tailoring learning experiences to individual needs, interests, and pace. Through adaptive content and interactive features, this model optimizes learning outcomes. The framework utilized an adaptive cognitive scaffolding mechanism based on real-time interaction data. As proficiency improved, scaffolding was gradually reduced through a process known as fading. For example, if a student’s mastery rate for the knowledge point of “Incoterms 2020” in Unit 5 was calculated at 42% (below the 60% passing threshold), the system paused progress to higher-order nodes (e.g., terms of pricing) and triggered an intervention loop. This intervention included the automatic generation of customized learning through remedial MOOC videos and targeted test items. Conversely, if the mastery exceeds 80%, the system triggers fading by reducing visual cues and explicit logical prompts, thereby fostering independent expertise.

*Step 4*: Closed-loop feedback and Optimization: This stage leveraged students’ learning behavior data, enabling teachers to dynamically recalibrate the correlation weights of knowledge points. By employing graph structure pruning and weight iteration algorithms, redundant nodes and weak correlations were systematically eliminated. Such continuous optimization ensures the robustness of graph logic while aligning instructional materials to the evolving demands of global industry.

This mechanism refined students’ personalized learning paths, facilitating progressive knowledge acquisition and improving instructional efficiency. By transcending the limitations of a traditional one-size-fits-all approach, it enables individualized education at scale. This personalized approach helps capture students’ interests, offering individual attention, and making learning relevant and engaging within their ZPD (Sayed et al., 2023). Moreover, the system established a data-driven “teach-learn-evaluate” closed-loop model, dynamically adjusting to student behaviors (e.g., dropout and relearning rates), to ensure a resilient and responsive educational ecosystem.

### 4.3 Evaluation: data analysis and effectiveness

#### 4.3.1 Data analysis method

The Evaluation phase utilized a single-group quasi-experimental design to assess the efficacy of the proposed model. In this study, the attainment of CO was quantified using the standardized calculation methodology mandated by the New Liberal Arts professional accreditation framework within the Yangtze River Delta region (Yao, 2025; Sun, 2023). As a pivotal metric for evaluating graduation competencies, CO aligns with the foundational principles of OBE: student-centeredness, goal-orientation, and continuous quality improvement. The evaluation matrix, presented in Table 1, employs a weighted distribution model to emphasize longitudinal learning processes and incremental competency acquisition. The formative component, contributing 40% to the final grade, functions as a comprehensive system that evaluates students’ pre-class preparation and in-class participation. Student progress is tracked through three main indicators: engagement rate in interactive classroom activities, completion rate of MOOC videos, and accuracy rate of test items on the Xueyin Online platform. The summative closed book exam constitutes the remaining 60% of the total grade, providing a high-stakes assessment of students’ higher-order synthesis of international trade knowledge and proficiency in English for Specific Purposes (ESP) developed throughout the instructional cycle.

The international trade practice course is designed to cultivate applied, innovative, and versatile professionals who possess the knowledge, skills, and competencies required by national vocational qualification certification required in careers related to international business (see Appendix 1 for detailed information). The evaluation of COs serves multiple purposes: it measures the effectiveness of the teaching model in enabling students to master core theoretical and practical knowledge on international trade practice, provides critical feedback for adjusting teaching strategies, and identifies limitations in the existing evaluation mechanisms to optimize them for better addressing individual learning discrepancies. The evaluation system allows teachers to identify students’ cognitive bottlenecks with high precision and functions through a closed-loop cycle. By analyzing real performance data, teachers can continually refine their educational practices and engage in reflective teaching to adapt the knowledge graph-based (KG-based) pedagogy to address learner-specific challenges effectively.

#### 4.3.2 Quantitative analysis of CO achievement

The achievement levels for total CO and individual sub-COs are determined through the evaluation components in Table 1, together with the following CO attainment formulas. The achievement rate is measured on a scale where scores between 0.7 and 0.84 indicate successful attainment, while scores exceeding 0.85 signify a shift toward higher-order cognitive mastery.

$$\text{Achievement level of}\;\mathrm{CO}=\frac{\text{Average score of the students}}{\begin{array}{c} \;\text{Total score of the comprehensive}\;\\ \text{evaluation of this course} \end{array}}$$

$$\begin{array}{l} \text{Achievement level of}\;\mathrm{sub}-\mathrm{CO}=\text{the}\;\mathrm{sum}\;\text{of the achivement}\;\\ \text{levels of assessment components}\;\;\;\text{that support the}\;\mathrm{CO}\;\text{in the}\;\\ \text{overall evaluation} \end{array}$$

The data presented in Table 4 indicate that during the 2024–2025 semester, students achieved an average CO attainment score of 0.78, demonstrating the strong success of the KG-based framework. Additionally, students performed better in the mastery of theoretical knowledge (CO1 = 0.81) and linguistic proficiency (CO2 = 0.82) compared to practical application (CO3 = 0.73). The disparity between conceptual mastery (CO1, CO2) and procedural execution (CO3) highlights the inherent limitations of declarative knowledge structures in supporting procedural mastery: while the KG effectively externalized implicit expert logic into a navigable semantic network, thereby reducing extraneous cognitive load, its primary function remains that of a conceptual map rather than a procedural simulator. The dual challenges posed by disciplinary complexity and the use of L2 medium of instruction were adequately mitigated to support schema construction. However, these issues persisted as bottlenecks for the automation of information in practical applications. The achievement score for CO3 underscores the pedagogical boundaries of the KG framework and suggests that high-order synthesis requires more than just adaptive scaffolding. Specifically, it demands haptic, repetitive interactions with industry-standard tools to facilitate procedural mastery.

*Performance analysis of CO1*: This objective pertains to fundamental theories and procedural frameworks in International Trade Practice. The high attainment rate of 0.81 demonstrates that KGs, as a semantic navigation tool, are capable of transforming fragmented trade rules (such as FOB, CFR, and 349 other entities) into logically coherent structures. The strong performance of students, reflected by classroom achievement rates of 92% and Xueyin online test scores of 90%, demonstrates that KG significantly alleviates the cognitive load associated with understanding complex disciplinary logic. Nonetheless, upon critical reflection on the evaluative architecture, an important downside is unveiled. Although theoretical underpinnings and procedural frameworks were well embedded in the evaluation, we are faced with something akin to a ceiling effect, where most individuals achieve generally high scores because the test questions are too easy. Although the theoretical foundations and procedural frameworks were effectively integrated, the evaluation’s low discriminative capacity limits its ability to differentiate students’ knowledge levels. For future iterations of teaching, it is recommended to utilize the difficulty adjustment features of the knowledge graph to design more challenging, unstructured questions, thereby enhancing the discriminative capacity of the evaluation.

*Performance analysis of CO2*: This objective was designed to integrate ESP proficiency with general adaptive and practical skills needed in global business settings. The achievement outcome of 0.82 demonstrates that the KG effectively reduces the linguistic challenges students face when processing specialized content by organizing terminology into semantic relationship networks. Consequently, most students have attained an acceptable proficiency level in oral and written English communicative skills required for import–export transactions. However, despite their competence in general foreign trade concepts, students continue to encounter difficulties in translating and applying specific technical terms. These challenges are evident from the low grade in term translation (61.8%) and the frequent inability to grasp the nuanced semantics necessary for professional accuracy in case analysis. The findings suggest that although the KG-driven adaptive scaffolding model effectively enhances students’ overall language abilities (Sarmiento-Campos et al., 2022), achieving expert-level terminological precision remains a crucial goal for improving pedagogy within the OBE framework.

*Performance analysis of CO3*: This objective evaluates the fundamental operational competencies required at various stages of international trade. The lowest achievement level (0.73) reveals that although KG-driven adaptive scaffolding supports the internal logic of trade operations, it encounters challenges in addressing higher-order procedural knowledge, such as letter of credit review and cost accounting. This finding underscores the importance of the OBE principle of Continuous Quality Improvement (CQI). While KG excels at externalizing declarative knowledge, future iterations should incorporate Digital Twin simulations to bridge the final gap between cognitive acquisition and professional readiness. By replicating trade procedures within a dynamic global trade environment, Digital Twin simulation tools enable learners to move beyond conceptual understanding and foster the development of practical operational skills.

### 4.4 Reflection: pedagogical implications and continuous quality improvement

The limited body of linking student learning outcomes to instructional approaches does not alter the fact that student achievements remain the central focus of the OBE framework. Student achievement evaluation transcends its role as a final assessment tool. It facilitates forward planning and enhances the precision of teaching practices, ultimately leading to improved teaching quality and sustainable cycles of teaching development. A quantitative analysis of CO achievement data revealed a discrepancy between performance levels and expected targets, emphasizing the need for targeted improvement measures in subsequent phases of teaching implementation. From a pedagogical perspective, these findings highlight the importance of conducting a systematic review of existing instructional strategies and evaluation mechanisms to ensure ongoing enhancement of educational quality.

1. Refined hierarchical evaluation

A test paper of moderate difficulty serves to distinguish candidates with varying levels of knowledge mastery, while a well-designed weighting system ensures a scientific and equitable distribution of scores across different difficulty levels. By differentiating the complexity and corresponding weight of test items, evaluations are better equipped to assess students’ actual learning proficiencies, thereby enhancing the accuracy and effectiveness of assessment practices.

The evaluation architecture adopts a tiered difficulty model segmented into foundational knowledge, applied professionalism, and extension and synthesis, comprising 30, 50, and 20% of the total evaluation weight, respectively. Foundational knowledge focuses on essential competencies, grounding the evaluation in core import–export protocols and fundamental conceptual frameworks. It establishes a baseline academic requirement that all students must meet. Applied professionalism, constituting the majority of the evaluation, represents core disciplinary content and emphasizes the application of knowledge in the context of real-world complexities. For instance, students are required to complete test items assessing their ability to navigate international trade scenarios, such as calculating the export price for products of high-tech industries and products of labor-intensive industries under the 2025 export tax refund updates^6^. Finally, extension and synthesis tasks measure students’ ability to integrate comprehensive knowledge of international trade practice to address complex trade issues. For example, test items involving the drafting or critical review of international trade documentation assess the application of knowledge related to sales contracts, terms of commodities, trade terms, price calculation, insurance, transportation in international trade operations to handle complex trade issues. This hierarchical evaluation system eliminates structural ambiguity in assessment frameworks, providing a clear basis for identifying learning gaps and ensuring that final grades authentically mirror students’ cognitive mastery.

1. Lexical precision interventions in business English

To address the challenges associated with ESP terminology deficiencies, we propose a dual-track pedagogical model that combines terminology analysis during class sessions with contextual translation exercises out of class. This approach transcends traditional rote memorization by embedding ESP terminology within authentic usage contexts, thereby fostering deeper understanding and long-term retention of specialized vocabulary. Classroom instruction focuses on distinguishing subtle differences between seemingly interchangeable terms, such as FOB v.s. CIF, facilitated by a digital textbook published by the course instructor. Vocabulary learning is transformed into active learning experiences through distinction-based exercises which enable students to use terminology as practical tools for trade operations. Role-play activities immerse students in buyers and sellers scenarios, allowing them to refine their negotiation skills by resolving price disagreements when applying trade terms in practice. Additionally, lexical competence is strengthened through performance in business communications simulating real-world international trade operations, such as correspondence negotiations, insurance claim negotiations, and the rigorous inspection of Letters of Credit. To further support classroom instruction, we implement biweekly digital “terminology check-in tasks,” which provide real-time feedback to correct fossilized language errors before they become ingrained in students’ professional practices.

1. Synergizing KG with digital twin

To address cognitive bottlenecks inherent in complex trade operations, subsequent iterations of this framework will integrate Digital Twin (DT) simulations with the established KG architecture. While the KG serves as the repository for structural declarative knowledge, the DT environment introduces a high-fidelity, synchronized manifestation of stochastic market volatility. This integration enables a pedagogical transition from static scaffolding to dynamic consequence navigation. Through the Automated Logic Validator, KG-based logic is applied to DT operational nodes, offering students with real-time, algorithmic feedback during risk-free, iterative simulations. This design establishes a bidirectional continuous quality improvement mechanism, wherein performance analytics generated within the DT environment inform recursive KG refinements. The resulting synergy ensures that the digital-intelligent framework dynamically addresses specific learning deficiencies, fosters robust professional competencies, and aligns with evolving industry demands.

## 5 Conclusion

Adopting the Educational Design Research paradigm, this study successfully developed and validated an adaptive cognitive scaffold powered by KG technology within an OBE-aligned CLIL environment. This study extends the application of CLT to the complex, interdisciplinary contexts of “New Liberal Arts”. We argue that the traditional “cognitive-language dualism” in CLIL can be mitigated through semantic externalization. By transforming implicit expert reasoning into navigable semantic networks, the KG-driven scaffold effectively “offloads” extraneous cognitive load. This process ensures that learners remain within their ZPD without triggering the expertise reversal effect, thereby refining our theoretical understanding of how AI-integrated tools can bridge the gap between intended learning outcomes and actual cognitive acquisition.

The findings successfully address the primary research objectives, demonstrating that the proposed model effectively alleviates dual cognitive bottlenecks and enhances holistic learning outcomes. During the development phase, it was evidenced that domain-specific KGs can externalize implicit expert logic into navigable semantic networks, while the quasi-experimental evaluation demonstrated that the adaptive scaffolding system significantly reduces the bipartite cognitive strain associated with language and content integration. Furthermore, the multidimensional evaluation substantiated the model’s efficacy in improving both conceptual understanding and linguistic proficiency, thereby effectively bridging the gap between intended learning outcomes and actual cognitive acquisition.

As a critical component of the OBE framework, the success of this intervention establishes a solid empirical foundation for developing a scalable “digital-intelligent” instructional model. The core pedagogical implication is that educators should prioritize the structured externalization of knowledge over simple content delivery. Through the use of KGs that organize domain knowledge into directed labeled graphs, instructors can provide students with a “semantic roadmap,” promoting learner autonomy and deeper conceptual understanding. This framework serves as a reproducible model applicable to other interdisciplinary domains where professional expertise and linguistic proficiency need to be cultivated simultaneously. While KGs demonstrated exceptional efficiency in conceptual scaffolding (CO1 = 0.81; CO2 = 0.82), this study highlights several critical pedagogical boundaries. Specifically, the persistent “knowledge-action gap” in procedural mastery (CO3 = 0.73) indicates that declarative knowledge structures alone are insufficient for cultivating high-order operational skills. This limitation underscores a haptic shortcoming in current KG-based interventions. Consequently, the study recommends integrating Digital Twin simulations into future instructional designs. Combining the structural logic of KGs with the dynamic, high-fidelity environments offered by DTs creates a synergistic potential, bridging the gap between cognitive acquisition and professional readiness.

Despite these promising results, several limitations must be acknowledged. First, due to the finite availability of elective course students, the study utilized a single-group quasi-experimental design. The absence of a control group limits the capacity to isolate the specific effects of the KG intervention from confounding variables, such as instructor influence. Second, the sample (*n =* 51) was drawn from a single application-oriented institution, which may introduce demographic bias and limit the generalizability of the findings across different proficiency levels or institutional tiers. Finally, reliance on platform-generated algorithmic data and specific OBE metrics may be susceptible to student platform behaviors. Furthermore, the observed high attainment rates suggest a potential “ceiling effect,” indicating that certain assessment items lacked sufficient psychometric difficulty to effectively discriminate between high-proficiency learners. Future research should adopt rigorous quasi-experimental designs incorporating control groups and multi-institutional longitudinal trials with larger cohorts. Additionally, incorporating psychometric assessments for critical thinking and innovation, complemented by qualitative phenomenological data, will further validate the efficacy of KG-based pedagogical interventions.

In summary, this research contributes to the Digital Humanities discourse by providing a theoretically grounded and technologically advanced KG-integrated CLIL paradigm, serving as a replicable model for cultivating interdisciplinary professionals in an increasingly digitalized educational landscape.

## Data Availability

The original contributions presented in the study are included in the article/Supplementary material, further inquiries can be directed to the corresponding author.

## Footnotes

1 The Test for English Majors-Band 4 (TEM-4) is a national standardized examination in China designed to rigorously assess the communicative competence and linguistic synthesis skills of English majors, in alignment with the “National Quality Standards for Foreign Language and Literature Education in Higher Education Institutions”.

2 POCIB is online simulation platform for comprehensive international trade skills training, has been developed by Nanjing Desunsoft Co., Ltd. It provides a simulated environment for enhancing practical international trade competencies, fostering professional skills, and supporting career development.

3 https://www.xueyinonline.com/ Xueyin Online platform is a innovative open learning MOOC platform co-developed by Chaoxing Group and the Open University of China. The platform serves as a national evaluation system which operates high-quality online courses for higher education, vocational training, and lifelong learning in China. Based on the credit bank concept, it offers a new educational structure and learning method for social learners, characterized by “lifelong learning,” “ubiquitous learning” and “future learning.” Moreover, it aids in building a learning society through constructing personal lifelong learning files.

4 The CVI serves as a psychometric metric to quantify the degree of alignment between measurement instruments, such as curriculum designs, assessment systems, and knowledge graphs, and their target domains in terms of content coverage, logical structure, and conceptual definitions.

5 I-CVI means item-level CVI and represents the proportion of experts endorsing relevance for a specific item. S-CVI/Ave means scale-level CVI and represents the average of the I-CVIs for all items. A denotes the number of experts rating an item as 3 or 4, and N is the total number of experts.

6 The tax rebate rate for products of high-tech industries maintains 17% while tax rebate rate for products of labor-intensive industries reduced to 11–13% under “Export Tax Rebate Rate Library 2025 Edition A” released by the State Taxation Administration in China on March 5, 2025.
