# Supplementary material for: Knowledge graph as an adaptive cognitive scaffolding: enhancing learning outcomes in CLIL-based international trade practice course under the OBE framework
Source: Front Psychol. 2026 May 22;17:1802856. doi: 10.3389/fpsyg.2026.1802856 (PMC13236954; doi:10.3389/fpsyg.2026.1802856)
Supplement: Supplementary file 1 [file Supplementary_file_1.docx]

**Course Objectives for International Trade Practice**

**Overall CO**: The primary goal of this course is to cultivate interdisciplinary foreign business professionals well-versed in foreign languages and international trade, aligning with the national vocational qualification requirements for knowledge, skills, and competencies related to international trade-related professions. This is achieved by integrating the applied educational orientation of the university and highlighting the “Foreign Language+” feature of the new liberal arts. According to the talent cultivation plan, curriculum standards, professional standards, and analysis of student needs, the course aims to develop “applied, innovative, and interdisciplinary” global foreign language talents with both Chinese affinity and an international perspective. Students who study International Trade Practice in a CLIL educational context will develop international perspectives while they learn about worldwide and domestic matters and build their ability to think creatively and learn independently, and develop global understanding. The students will develop their professional competencies and practical skills through hands-on instruction which teaches them to understand international trade laws and develop their ability to evaluate worldwide business risks and handle trade-related conflicts and reduce business transaction risks.

**CO 1**: Students will learn about all the theoretical elements and operational aspects of international trade import and export activities through this course. This encompasses the complete workflow of international trade import and export activities, including business negotiation processes, international trade terminology, international trade sales contracts execution, along with both theoretical and practical knowledge about goods classification, quality standards, quantity needs, packaging and marking systems, pricing methods, settlement procedures, transportation options, insurance coverage, claims handling, and commodity inspection.

**CO 2**: The CLIL education program helps students improve their English language abilities, cultivate cross-cultural capabilities and adaptability through international trade business education and develop flexible work habits and gain practical international trade skills. The course requires students to master essential English expressions which relate to trade theories and policies while they study fundamental foreign trade concepts and policy documents and develop basic communication competencies for writing business letters and conducting import-export operations through oral discussions, which help them achieve successful business English interactions.

**CO 3**: This CO requires students to develop practical competencies throughout all phases of international trade import-export operations. This encompasses proficiency in the utilization of international settlement instruments under multiple payment methods; the ability to handle letters of credit through their issuance and modification and verification process; the aptitude to compile complete documentation packages in accordance with sales contracts, letters of credit, international customs rules, all applicable laws and regulations; and the ability to conduct cost calculations and quotation adjustments, freight charge calculations, commission and discount computations, and exchange rate conversions in the context of international trade.
